# Supplementary material for: Structural analysis of 70S ribosomes by cross-linking/mass spectrometry reveals conformational plasticity
Source: Sci Rep. 2020 Jul 28;10:12618. doi: 10.1038/s41598-020-69313-3 (PMC7387497; doi:10.1038/s41598-020-69313-3)
Supplement: Supplementary file 1 — Supplementary Information [file 41598_2020_69313_MOESM1_ESM.docx]

**SUPPLEMENTARY INFORMATION**

**Structural Analysis of 70S Ribosomes by Cross-Linking/Mass Spectrometry Reveals Conformational Plasticity**

Christian Tüting^1+^, Claudio Iacobucci^2,$+^, Christian H. Ihling^2,3^,

Panagiotis L. Kastritis^1,4,5*^, and Andrea Sinz^2,3*^

^1^Interdisciplinary Research Center HALOmem, Charles Tanford Protein Center, Martin Luther University Halle-Wittenberg, Kurt-Mothes-Str. 3a, 06120 Halle/Saale, Germany

^2^Department of Pharmaceutical Chemistry & Bioanalytics, Institute of Pharmacy, Charles Tanford Protein Center, Martin Luther University Halle-Wittenberg, Kurt-Mothes-Str. 3a, 06120 Halle/Saale, Germany

^3^Center for Structural Mass Spectrometry, Charles Tanford Protein Center, Martin Luther University Halle-Wittenberg, Kurt-Mothes-Str. 3a, 06120 Halle/Saale, Germany

^4^Institute of Biochemistry and Biotechnology, Martin Luther University Halle-Wittenberg, Kurt-Mothes-Str. 3, 06120 Halle/Saale, Germany

^5^Biozentrum, Martin Luther University Halle-Wittenberg, Weinbergweg 22, 06120 Halle/Saale, Germany

^$^current address: Corporate Preclinical R&D, Analytics and Early Formulations Department, CHIESI FARMACEUTICI S.p.A., Via Palermo 26/A, 43122 Parma, Italy

^+^Both authors contributed equally to this work

^*^Corresponding authors: Andrea Sinz, [andrea.sinz@pharmazie.uni-halle.de](mailto:andrea.sinz@pharmazie.uni-halle.de)

Panagiotis L. Kastritis, [panagiotis.kastritis@bct.uni-halle.de](mailto:panagiotis.kastritis@bct.uni-halle.de)

**Supplementary Methods**

**DSAU chemistry.** Considering range in spacer lengths of amine reactive, urea-based MS-cleavable cross-linkers, DSAU stands between DSBU and CDI. DSAU has four carbon atoms less than DSAU and four more than CDI (Scheme 1A). Its C2 spacer arms are the shortest possibility to connect the central MS-cleavable urea moiety (Scheme 1B) and the NHS reactive head groups. DSAU can bridge residues within Cα-C Cα distances of ~25 Å increasing the spatial resolution of DSBU by ~16%, while maintaining the reactivity of all NHS esters-based cross-linkers. Compared to CDI, DSAU is longer, but more selective towards lysine residues. In fact, despite its symmetric structure, CDI is not a homobifunctional cross-linker. Its first imidazole moiety is highly reactive and readily substituted by hydroxy (serines, threonines, and tyrosines) and amine groups (lysines, *N*-termini) in proteins. The resulting intermediate is considerably stable and the second imidazole is slowly displaced almost exclusively by primary amines. The high polarity of the urea moiety, combined with the shorter aliphatic chains of the spacer, makes DSAU less soluble in organic solvents than DSBU and CDI. In our hands, DSAU was solubilized in neat acetonitrile (ACN) up to 5 mM by sonication. This limits the final concentration of DSAU in the reaction mixture. To keep the ACN concentration below 2% (v/v) in the protein buffer, the concentration of DSAU will be 100 µM. More concentrated DSAU stock solutions might be prepared by replacing ACN with hexafluoroisopropanol. Alternatively, suspensions of 20-30 mM DSAU can be pipetted into ACN and then added to the protein mixture.

DSAU was synthesized following the same procedure of DSBU and using glycine as starting material instead of γ-aminobutyric acid (Scheme 1C). DSAU is also commercially available and was purchased from CF Plus Chemicals.

**
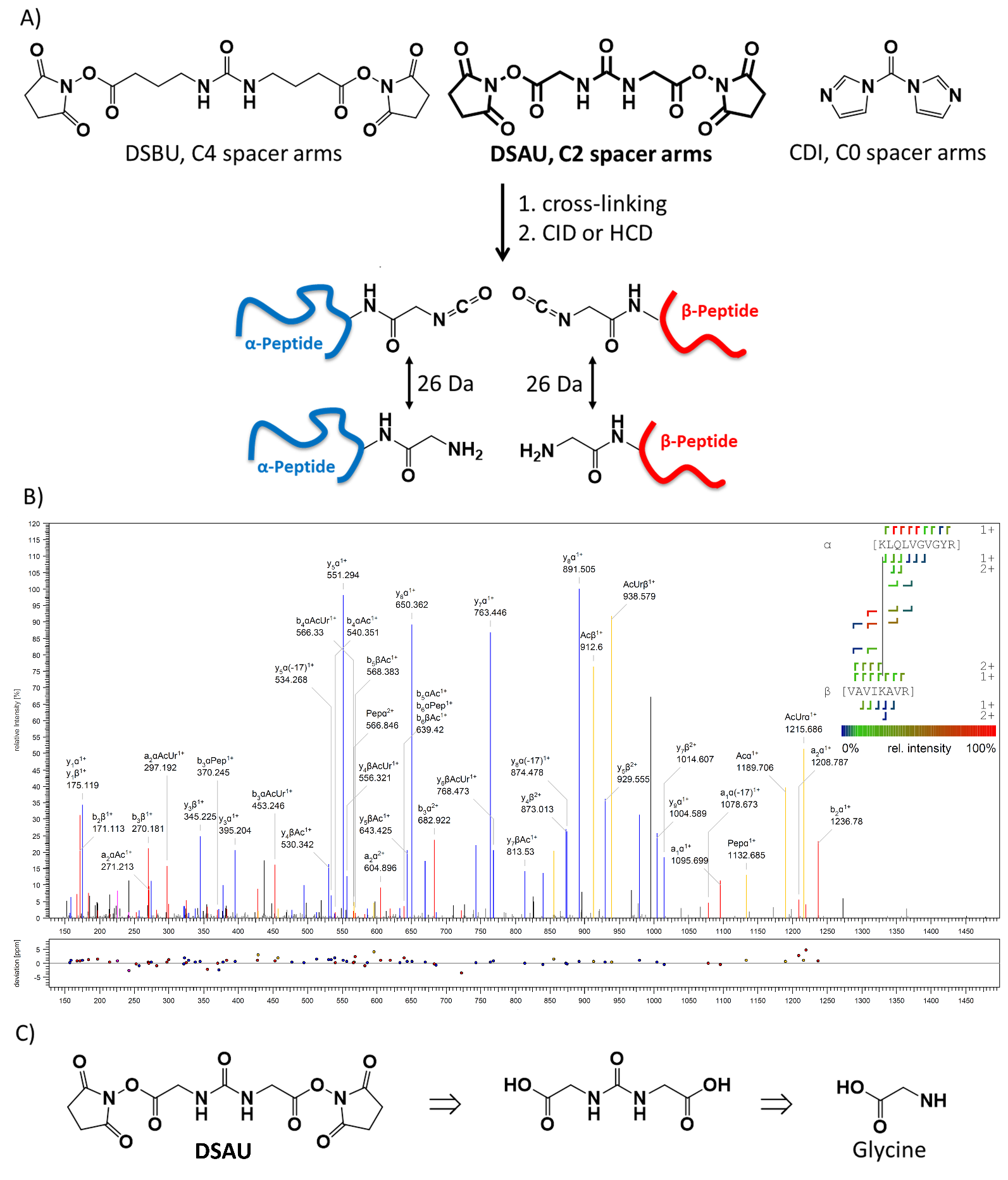
**

**Scheme 1.** A) Structures of the amine reactive, urea-based MS-cleavable cross-linkers disuccinimidyl dibutyric urea (DSBU), diacetyl dibutyric urea (DSAU) (highlighted in bold), and 1,1’-carbonyldiimidazole (CDI), exhibiting different spacer lengths. B) Fragment ion mass spectrum of a DSAU reaction product, assigned by MeroX 2.0 for a precursor ion at *m/z* 709.764, charge state 3+, corresponding to an intermolecular cross-linked product between K86 of RL6 and K71 of RL7. DSAU reacts mainly with amine groups. Upon fragmentation, DSAU yields two doublets (yellow signals) with a mass difference of 25.979 u in the MS/MS spectrum, facilitating automated data analysis. C) Retrosynthetic pathway of DSAU.

**Supplementary Figures**


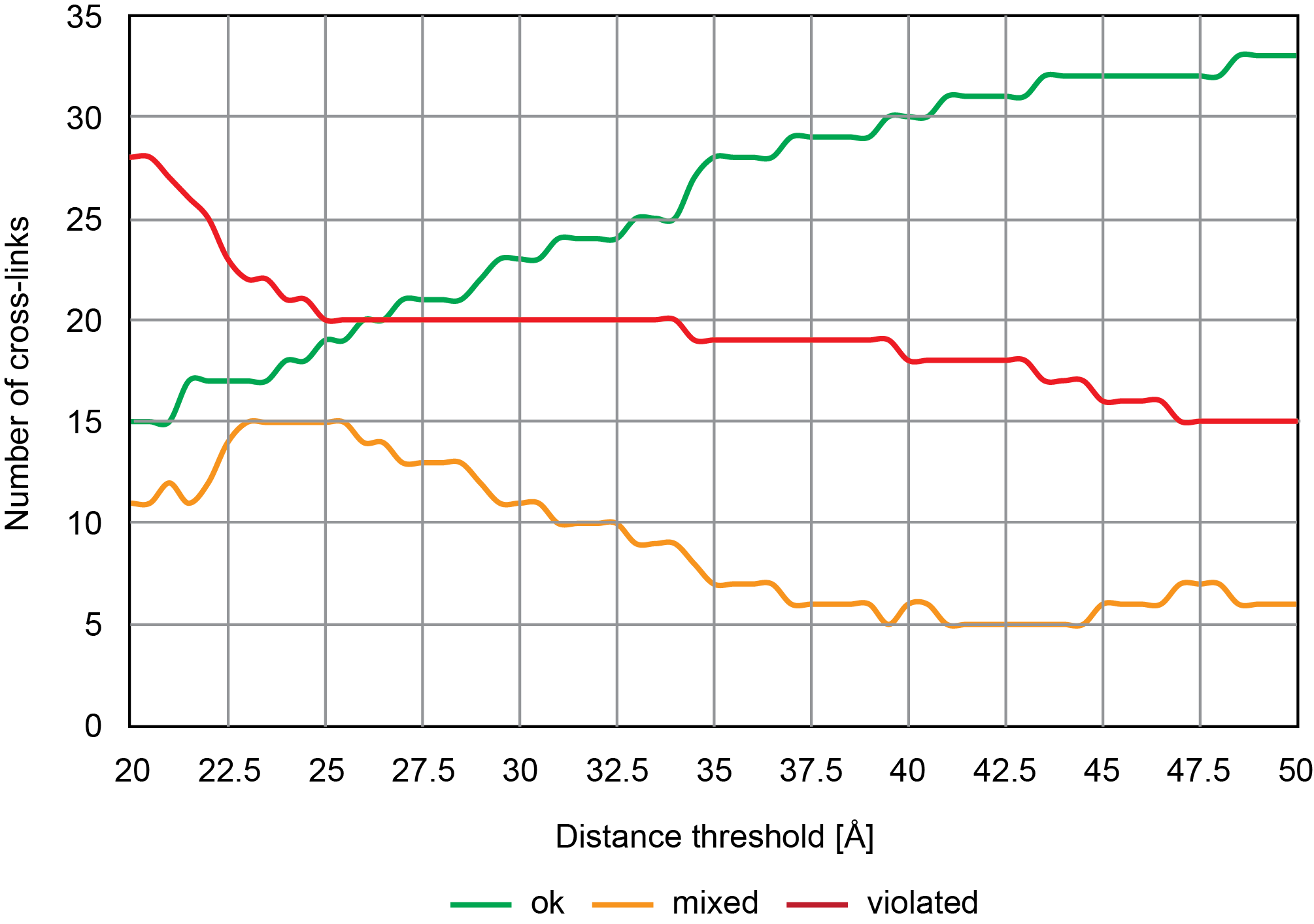


**Figure S1. Distribution of cross-link satisfaction and violation using different thresholds.** Cross-links are classified as ‘ok’, if they are satisfied for all identified structures and ‘violated’ when they are always above the threshold. ‘Mixed’ cross-links showed both, satisfaction and violation, for different structures indicating flexibility in the respective regions of the *E. coli* ribosome.


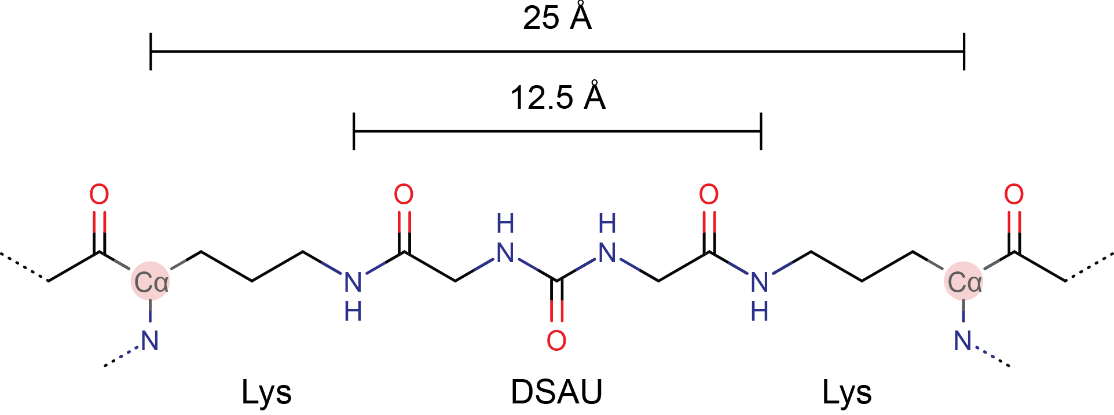


**Figure S2. Schematic view of a Lys-Lys cross-link generated by DSAU.** The maximum Cα- Cα distance between the connected lysines is 25 Å.


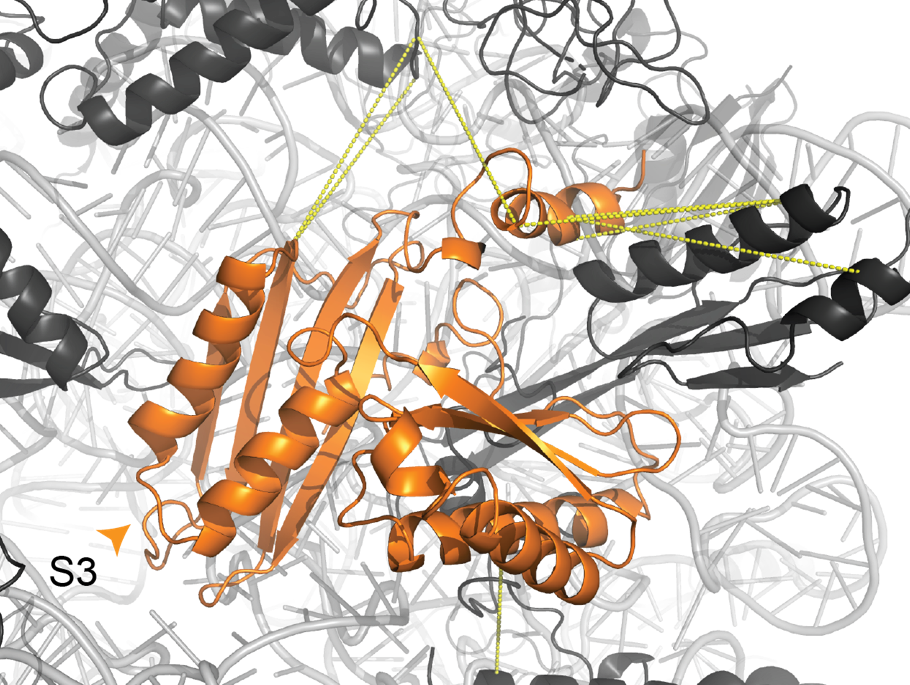


**Figure S3. 3D Model of S3 satisfying all cross-links without cross-link driven remodeling.** (A) Structural model of S3 (orange) after reconstruction of the *C*-terminal region. Ribosomal proteins (black) and rRNA (grey) are shown in cartoon presentation. Cross-links are shown as yellow dotted lines. The image was generated with PyMOL (Version 2.3.2), Schrödinger, LCC. URL: https://pymol.org.


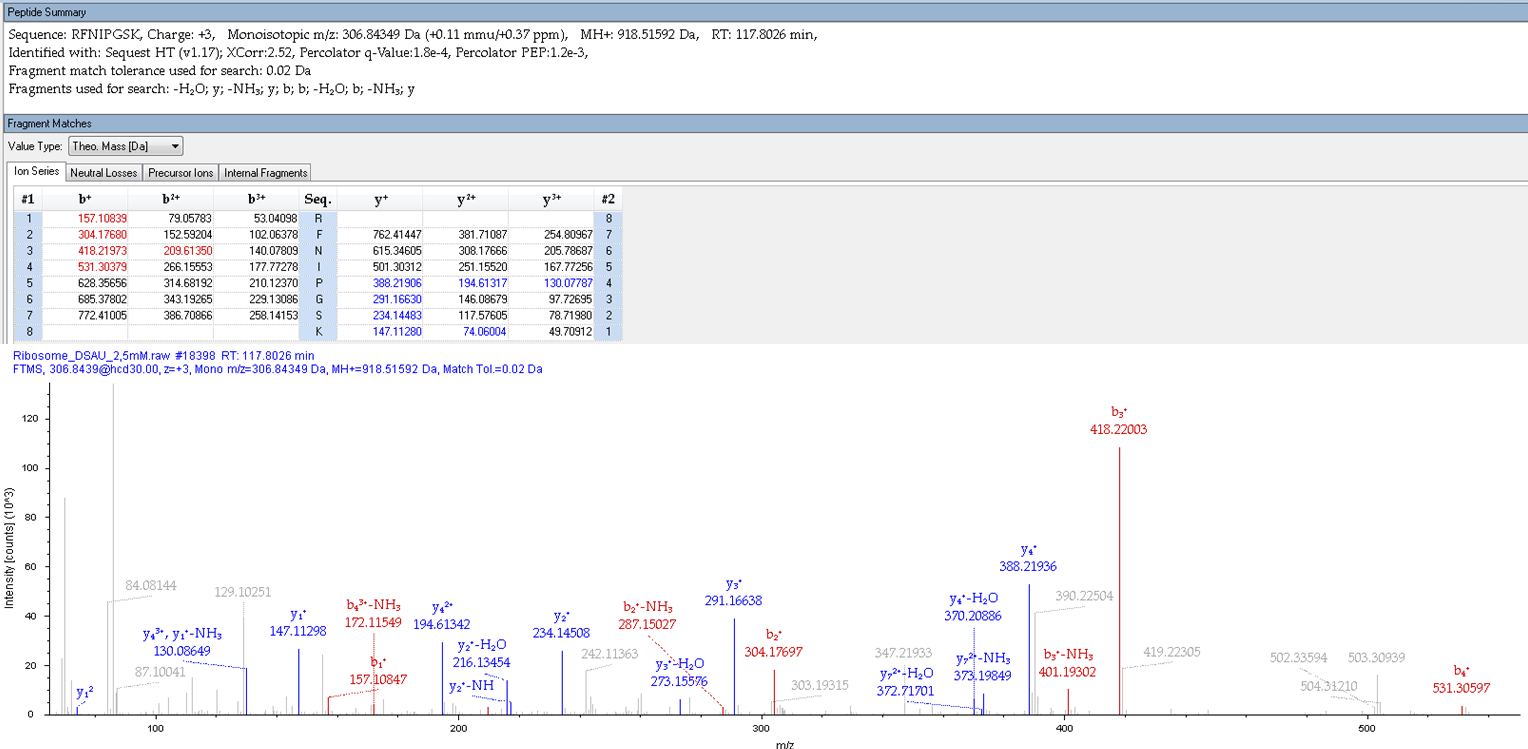


**Figure S4.** Fragment ion mass spectrum of the C-terminal peptide of L31 (amino acids 62-70, RFNIPGSK). The spectrum was annotated by the Sequest HT algorithm in Proteome Discoverer 2.4.


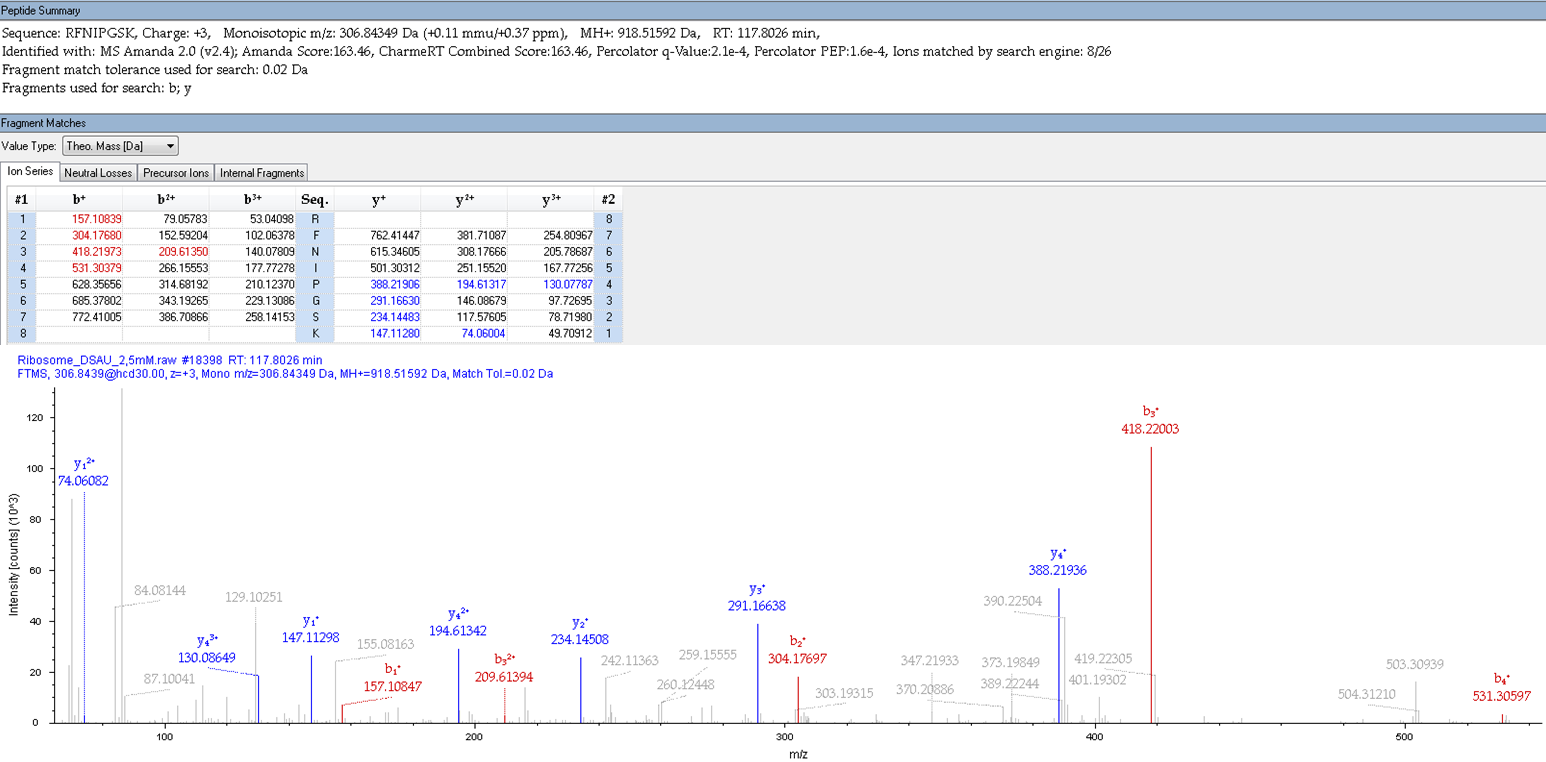


**Figure S5**. Fragment ion mass spectrum of the C-terminal peptide of L31 (amino acids 62-70, RFNIPGSK). The spectrum was annotated by the Amanda 2.0 algorithm in Proteome Discoverer 2.4.

**Supplementary Tables and Legends**

**Table S1. Summary of cross-link pairs.** Intermolecular cross-links are marked with “#”, intramolecular cross-links with “§”.

**Table S2. Summary of intramolecular cross-links.** PDB ID, chain identifier, amino acid numbers, the distances (in Å) are given for cross-linked residues.

**Table S3. Summary of intermolecular cross-links.** PDB ID, chain identifier, amino acid numbers, the distances (in Ångstrom) are given for cross-linked residues.

**Table S4. Intra-molecular cross-link mapping of L7/L12.** Cross-links were evaluated in monomeric or multimeric models with Xwalk [1].

**Table S5. Summary of cross-links for the final model.** Cross-link IDs are annotated according to Table S1. Protein name and UniProt Identifier are given. Distances are given in Å and cross-links that could not be mapped are indicated (n.m.). The distances are classified as follows: Satisfied (<37.5 Å for inter-, <30 Å for intramolecular cross-links), minor violated (<50 Å for inter-, <40 Å for intramolecular), and violated. The classification from large-scale mapping is given in brackets.

**Supplementary References**

1. A. Kahraman, L. Malmstrom & R. Aebersold. Xwalk: computing and visualizing distances in cross-linking experiments. *Bioinformatics* **27**, 2163-2164, doi:10.1093/bioinformatics/btr348 (2011).
